# Mostafa Maged Maneuver to Control Post-Partum Hemorrhage during Vaginal Delivery

**DOI:** 10.4314/ejhs.v33i3.9

**Published:** 2023-05

**Authors:** Mostafa Maged Ali

**Affiliations:** 1 Obstetric and gynaecological Department, Ministry of Health, Fayoum General Hospital, Fayoum, Egypt

**Keywords:** Postpartum hemorrhage, Mostafa Maged maneuver, vaginal delivery, uterine compression, atony

## Abstract

**Background:**

This study is aimed to introduce a new technique used in controlling the postpartum bleeding during normal vaginal delivery.

**Methods:**

This study was conducted on 150 pregnant women aged 18 years or more who were eligible for normal vaginal delivery. After placental delivery, Mostafa Maged maneuver was applied. The amount of blood loss was estimated by counting the number of pads immediately before applying the maneuver compared to the number of pads immediately after applying the maneuver to determine the effectiveness of the Mostafa Maged maneuver.

**Results:**

The study revealed that 15.3% of the study group had atony, and 23.3% were complicated with postpartum hemorrhage. In terms of blood loss, the average number of pads used before Mostafa Maged compression was (2.8) pads, with a range of 1 to 6 pads. The mean blood loss volume was (353.3±158.5) ml ranging between 125 and 750 ml. we should consider that Mostafa Maged maneuver is immediately applied after placental delivery. That is why small amount of 125 ml of blood loss in few patients is estimated, which decreased to (0.54±0.14) pads ranging between 0.5 and 1 pad with a mean (65.2±17.7) ml blood loss ranging between 60 and 125 ml after applying Mostafa Maged maneuver during normal vaginal delivery. There was a statistical decrease in the number of pads and volume of blood loss after Mostafa Maged maneuver.

**Conclusion:**

Mostafa Maged maneuver was found to be effective in stopping and preventing postpartum bleeding during vaginal delivery as this maneuver is used as a prophylaxis against post-partum hemorrhage in patients with risk factors of postpartum hemorrhage. It is also used to control post-partum hemorrhage during vaginal delivery. It has been proven to be tolerable, easy to learn and easy to perform.

## Introduction

This study is aimed to introduce a new technique used in controlling the postpartum bleeding during normal vaginal delivery. Postpartum hemorrhage is the leading cause of maternal deaths worldwide.

Postpartum hemorrhage is the most catastrophic complication of childbirth. It is defined as blood loss of more than 500 of blood during vaginal delivery (1,16). The resuscitation of patients is the main target of all obstetricians to control and manage postpartum bleeding.

Controlling the patient's hemodynamic condition is mandatory for doctors to protect the patients from the complications of shock status; consequently, intravenous fluids or blood packets can be administrated to compensate for the blood loss after delivery (2).

No uterine contractions, clinically defined as atony, may result in excessive blood loss. Uterotonics induce and enhance contractions of the uterus to prevent atony and accelerate placental delivery. Obstetricians are familiar with uterotonics such as oxytocin, ergometrine, misoprostol and cabertocin (3).

The first method of controlling postpartum bleeding due to atony is bimanual compression. Uterotonics can also be used for controlling bleeding during labour plus bimanual compression, which showed significant effectiveness in controlling postpartum haemorrhage (3,4). When uterine atony occurs, there are many medical types of equipment (e.g., Bakri SOS tamponade balloon catheter, Sengstaken Blakemore esophageal catheter, Foley catheter filled with 60 to 80 mL of sterile solution, Rusch hydrostatic urologic balloon, Hydrostatic condom catheter, and Uterine packing) can be placed inside the uterine cavity to ensure that compression of uterine walls is applied (3).

Uterine compression sutures, such as B-lynch, Mostafa Maged and Cho and Hayman, have a more significant effect on uterine preservation than hysterectomy. All techniques involve the application of sutures into and over the uterus to control bleeding (5,6).

Mostafa Maged maneuver is an easy technique used in normal delivery and a simple maneuver used primarily in the atonic uterus in postpartum immediately after delivery of the placenta (7). This study is aimed to introduce Mostafa Maged maneuver for stopping and preventing postpartum bleeding during vaginal delivery.

## Patients and Methods

All relevant information, like the purpose and methodology of the experiment, was explained to study participants beforehand, and informed consent was obtained. All procedures followed were in accordance with the ethical standards of the responsible committee on human experimentation (institutional and national) and with the Helsinki Declaration of 1975, as revised in 2008. This trial was registered on www.clinicaltrials.gov under clinical trial number is (NCT05288322).

This study has been granted the ethical committee approval from a university in Egypt as described with IRB (R 218). A sample size of 150 cases was calculated by epi info 2000 software based on prevalence of outcome in the previous published case-series study at confidence interval 95% and 80% power of the study. The sample is increased by 90% to overcome missing data. The data that support the findings of this study are available on request to the corresponding author. The data are not publicly available.

This study was conducted on 150 pregnant women aged 18 years or more who were eligible for vaginal delivery. All participants were subjected to a detailed history and a general examination to exclude the presence of any disorders such as hypertension, diabetes, collagen diseases, kidney or liver diseases and hematological diseases. Obstetric examinations were performed according to each center's protocol.

The inclusion criteria were pregnant females aged from 18 to 40 years old.

The exclusion criteria were: smokers, morbidly obese patients, and who have a medical history of blood and collagen diseases

Although physicians have their own protocol to manage postpartum bleeding, we did not use it to manage postpartum hemorrhage. All doctors' protocol includes: First, adding uterotonics, and if it failed, we proceed to use the Bakri balloon. Then, surgical procedures were applied, such as cho sutures, and other compression sutures and hysterectomy, if all measures failed. After placental delivery, the new maneuver was done by Mostafa Maged for the entire four minutes (100% of patients) with adding uterotonics as oxytocin 5 mg intravenously.

**Figure 1:**
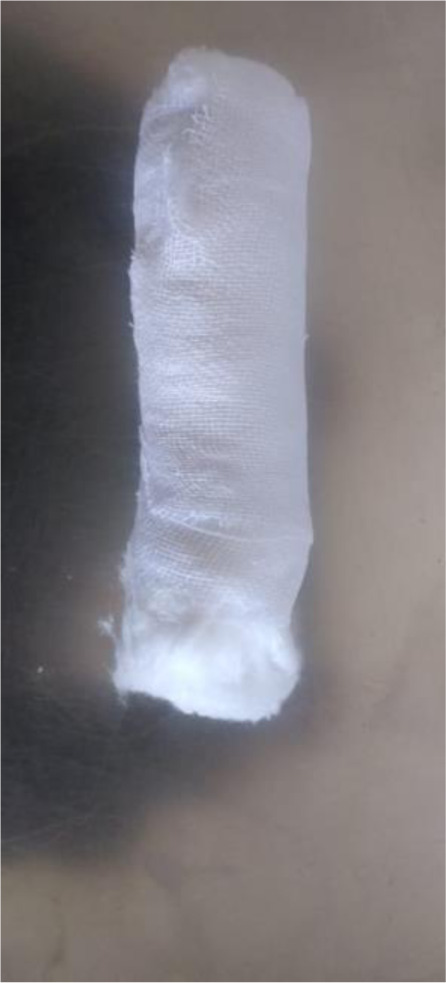
Pad used in the study to calculate the blood loss amount (15 cm*3 cm*4 cm).

The amount of blood loss was estimated by comparing the number of pads immediately before and after applying the maneuver (one pad can tolerate 125 ml of blood; this volume of blood in one pad is calculated by weighing the pad before and after being soaked by blood)) to determine the effectiveness of Mostafa Maged maneuver. The pad used in the study is revealed in Figure (1)

The pad measures (15 centimetre by 4 cm by 3 cm). The obstetrician changes the pads then they are gathered and weighed with the understanding that one milligram of the pad's weight equals one ml. of blood.

All subjects were followed up for six hours to detect if there was any occurrence of postpartum bleeding.

**Figure 2:**
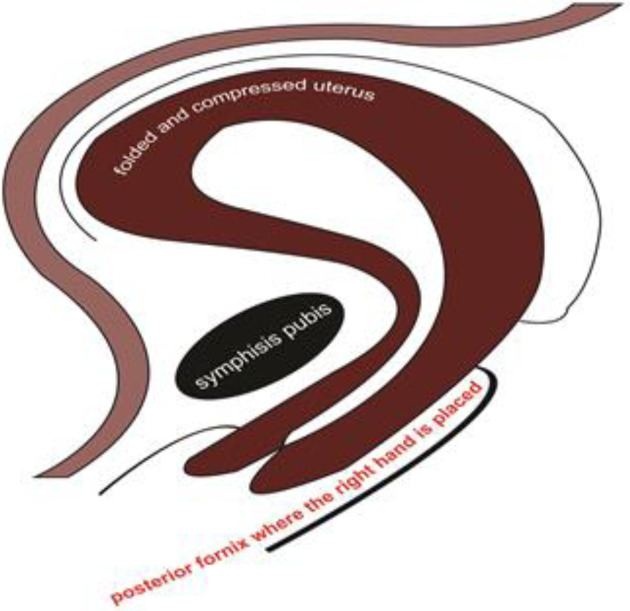
Mostafa Maged maneuver.

**Procedure**: The first step in the Mostafa Maged maneuveur step is placing the right hand to the posterior fornix of vaginal canal trying to put pressure on the cervix and the lower part of uterus compressing the anterior and posterior walls of the lower uterine segment. The second is placing the left hand over the fundus of the uterus and the posterior wall of the uterus from the abdominal part of the pregnant mother (the side of the abdominal skin). The third step is trying to grasp the whole uterus by the two hands abdominally and vaginally against the symphysis pubis as if the uterus is containing or surrounding the symphysis pubis bone, and in this way, getting the anterior and posterior walls of the uterus against each other (compression achieved) and reducing the intra-cavitarian space so prevention of the expansion of the uterus is applied. Also, with our right hand, we seal the outlet of the genital tract, so we arrest the blood coming out of the uterus allowing the hemostasis process to be achieved. It is illustrated in Figures 1 and 2.

**Statistical analysis**: Data was collected and coded to facilitate data manipulation and double entered into Microsoft Access, and data analysis was performed using the Statistical Package of Social Science (SPSS) software version 22 in windows 7 (SPSS Inc., Chicago, IL, USA). Simple descriptive analysis in the form of numbers and percentages of qualitative data, arithmetic means as central tendency measurement and standard deviations as a measure of the dispersion of quantitative parametric data. Independent samples t-test was used to compare quantitative measures between two independent groups. Paired t-test was used to compare two dependent quantitative data.

For qualitative data, the Chi-square test was used to compare two or more than two qualitative groups. Multiple linear regressions were used to test the association between quantitative dependent and independent variables and the detection of risk factors. *P-value* < 0.05 was considered statistically significant.

## Results

Table 1 illustrates that the mean age of the study group was 27.4 [(6.9)], and the mean weight among the study group was 76.2 [(4.1)] kg, with a mean height of 169.3 [(3.5)] cm, while the mean BMI was 26.6 [(1.8)] kg/m2. For blood pressure, the mean systolic blood pressure was 107.7 [(13.7)], and the mean diastolic blood pressure was 74.4 [(8.2)].

**Table 1:** Description of age, anthropometric measures, and blood pressure among the study group and Medical history of the study group (n=150)

Variables	Mean (SD)	Range
**Age (years)**	27.4 (6.9)	18-40
**Anthropometric measures**		
**Weight (kg)**	76.2 (4.1)	69-85
**Height (cm)**	169.3 (3.5)	165-177
**BMI (kg/m^2^)**	26.6 (1.8)	23.8-31.2
**Blood pressure**		
**Systolic**	107.7 (13.7)	70-170
**Diastolic**	74.4 (8.2)	60-100-
**Gestational age (wk)**	37 (2)	27-40
	**Number**	**%**
**Parity**		
**Primi**	54	36%
**Multi**	96	64%
**Gestational age**		
**Preterm**	16	10.7%
**Full term**	134	89.3%
**Number of fetus**		
**Single**	54	36 %
**Twins**	96	64 %

According to Table 1, 36% of the study group were primigravida, whereas 64% were multipara. Regarding gestational age, 10.7% were preterm, while 89.3% were full term. For the number of the fetus, 97.3% had a single baby, while 2.7% had twins. The mean gestational age of 37.0 [(2)] weeks ranged between 27 and 40 weeks.

According to the results in Table 2, 36% of the study group underwent episiotomy, 15.3% had atony, and 23.3% had a postpartum hemorrhage. The average number of pads used before double hand compression to assess blood loss was (2.81.3) pads, with a range of 1 to6 pads. The mean blood loss volume of 353.3 [(158.5)] ml ranged between 125 and 750 ml, which decreased to 0.54 [(0.14)] pads ranging between 0.5 and 1 pad with mean 65.2 [(17.7)] ml blood loss ranging between 60 and 125 ml after Mostafa Maged maneuver was applied.

**Table 2:** Intra-operative assessment and outcomes of the study group (n=150)

Variables	Number	(%)
**Episiotomy**		
No	96	64%
Yes	54	36%
**Atony**		
No	127	84.7%
Yes	23	15.3%
**Postpartum hemorrhage**		
No	115	76.7%
Yes	35	23.3%
**Time of applying this maneuveur**		
Four minutes	150	100%
**Usage of uterotonics**		
Yes	150	100 %
No	0	0 %
**Blood loss assessment**		
**N.B : One pad can tolerate 125 ml of blood**	**Mean (SD)**	Range
No of pads before	2.8 (1.3)	1-6
No of pads after	0.54 (0.140	0.5-1
Blood loss volume (ml) before	353.3 (158.5)	125-750
Blood loss volume (ml) after	65.2 (17.7)	60-125

Table 2 shows a statistically significant decrease in the number of pads and volume of blood loss with a p-value <0.001 after applying the Mostafa Maged maneuver. According to Table 2, there is a statistically significant higher volume of blood loss with a p-value<0.001 before applying the Mostafa Maged technique in cases with PPH. In contrast, there was no difference between the two PPH groups regarding blood loss volume after applying the Mostafa Maged technique.

Tables (3, 4, 5) shows a statistically significant lower weight and BMI with a p-value of 0.01 in patients who were complicated with postpartum hemorrhage. In addition, there was a statistically significant association between atony and PPH as all cases of atony developed postpartum hemorrhage. On the contrary, there was no statistically significant effect on age, height, blood pressure, parity, gestational age, fetus number, or episiotomy on developing PPH.

**Table 3:** Comparisons of blood loss before and after applying the Mostafa Maged maneuver in the study group

Variables	Before		After		P-value
		
	Mean	SD	Mean	SD	
**Number of pads**	2.8	1.3	0.54	0.14	<0.001*
**Blood loss volume (ml)**	353.3	158.5	65.2	17.7	<0.001*

**Table 4:** Comparisons of blood loss before and after applying Mostafa Maged maneuver in PPH groups

Blood loss volume (ml)	No PPH (N=115)		PPH (N=35)		P-value
		
	Mean	SD	Mean	SD	
**Before**	275	64.6	610.7	84.5	<0.001*
**After**	64.5	16.6	67.4	20.9	0.4

**Table 5:** Risk factors in different PPH groups

Variables	No PPH		PPH		P-value
	(N=115)		(N=35)		
		
	Mean (SD)		Mean (SD)		
Age (years)	27.2 (6.5)		27.7 (8.2)		0.7
Weight (kg)	76.7 (4.03)		74.8 (3.9)		0.01*
Height (cm)	169.2 (3.7)		169.8 (2.8)		0.4
BMI (kg/m^2^)	26.8 (1.9)		25.9 (1.5)		0.01*
Systolic BP	106.4 (9.4)		111.7 (22.5)		0.2
Diastolic BP	74.3 (7.8)		74.9 (9.7)		0.7
**Parity**					
Primi	111	96.5%	35	100%	0.6
Multi	4	3.5%	0	0%	
**Gestational age**					
Preterm	14	12.2%	2	5.7%	0.4
Full term	101	87.8%	33	94.3%	
**Number of fetus**					
Single	42	36.5%	12	34.3%	0.8
Twins	73	63.5%	23	65.7%	
**Atony**					
No	115	100%	12	34.3%	<0.001*
Yes	0	0%	23	65.7%	

The multivariate logistic regression model analysis was conducted to explore the explanatory power of different risk factors in predicting blood loss volume. According to Table 6, there is a statistical significance prediction power with *a* P-value *< 0.05* to decrease diastolic blood pressure and occurrence of atony with no significance for other factors as shown in table 6.

**Table 6:** Multivariate linear regression analysis to determine the prediction power of different risk factors on the amount of blood loss

Variables	B	SE	Beta	Sig
Constant	-7568.3	7343.9	----	0.3
Age (years)	-1.2	1.2	-0.05	0.3
Weight (kg)	-41.9	48.2	-1.1	0.4
Height (cm)	4573.8	4356.8	1	0.2
BMI (kg/m2)	121.8	136.9	1.4	0.4
Systolic BP	1.4	0.73	0.12	0.06
Diastolic BP	-4.7	1.2	-0.25	0.001*
Gestational age (wk)	6.5	4.1	0.08	0.11
Number of fetus	71.1	50.7	0.07	0.2
Episiotomy	2.2	16.1	0.007	0.9
Atony	366.8	21.5	0.84	0.001*

## Discussion

Globally, maternal mortality remains unacceptably high, with up to 400 maternal deaths per 100,000 live births (8,15,16). The proposed method is due to the low level of economic development in many countries and the need to use the methods that do not require significant financial expenditures and time. I believe this study to be the first evaluating the impact of Mostafa Maged maneuver on management of severe PPH, showing an associated reduced need for surgical intervention.

Postpartum haemorrhage is a problem worldwide, especially in developing countries and low-resource clinics. Due to the low resources and inadequate facilities, those countries desperately need simple and tolerable techniques to manage those cases of postpartum bleeding (8). No severe postpartum hemorrhage occurred in this current study compared to a worldwide rate of 2.8% (95 % CI 2.4–3.2). Severe postpartum haemorrhage is defined as more than 1000 ml of blood loss in normal vaginal delivery (9).

I consider this study as a hypothesis-generating study for drawing an evidence-based conclusion for this Mostafa Maged maneuver. Mostafa Maged conducted only one case series study on ten patients and was effective on pregnant women (7). In comparison with other techniques, although bimanual uterine compression is referenced and taught in many training programs, the results of this study have revealed that the application of the technique by a single provider, even when correctly performed, may be inadequate to sufficiently compress the uterus for the recommended amount of time (10).

Individuals were unable to fully compress the uterus and maintain compression for more than 150 seconds without fatigue. Fatigue during applying Mostafa Maged technique is not felt as quickly as we feel during Regular bimanual uterine compression (10, 14).

Chad A GROTEGUT demonstrated that oxytocin exposure is a significant independent risk factor of severe PPH secondary to uterine atony, which is unpredictable. This finding supports the molecular mechanisms involved in oxytocin receptors desensitization in the setting of prolonged oxytocin desensitization leading to decreases in oxytocin-mediated function. Protocols that decrease the amount of oxytocin that patients receive may decrease the incidence of PPH secondary to uterine atony (11).

The primary outcome in the study of Minoo Rajaei was the amount of hemorrhage. The study of their's showed that mitotic is more beneficial in preventing PPH than oxytocin, which is consistent with the outcome of the study by Lokugamage et al., who reported the superiority of misoprostol over Syntometrine in controlling PPH. Other studies have shown better misoprostol efficacy than methylergometrine in preventing PPH (12,13).

The oral dose of misoprostol has an 8-minute onset of action, and the sublingual dose has an 11-minute onset of action and duration of action of approximately 3 hours. A vaginal dose has a 20-minute onset of action, so this delay in the onset of misoprostol action onset is avoided by applying Mostafa Maged maneuver in the immediate postpartum period.

Furthermore, this study was conducted at Duke University to determine the effectiveness of oxytocin on uterine atony incidence, and compared to the Mostafa Maged maneuver, this new maneuver is of great value. There are many side effects with those medications used in normal vaginal delivery.

In contrast to the Mostafa Maged maneuver, which has no side effects, oxytocin can cause hypotension and tachycardia; misoprostol can cause allergy and fever due to the effect of prostaglandin on the hypothalamus. However, Mostafa Maged maneuver is effective and easy to use. Mostafa Maged has proven a statistically significant decrease in the number of pads and volume of blood loss with a p-value <0.001, after applying the Mostafa Maged maneuver.

The Mostafa Maged maneuver demonstrated a statistically significant higher volume of blood loss with a p-value <0.001 before applying the Mostafa Maged technique in cases with postpartum bleeding. In contrast, there was no difference between two PPH groups regarding blood loss volume after applying the Mostafa Maged technique.

In conclusion, this study is aimed to introduce Mostafa Maged maneuver for stopping and controlling postpartum bleeding during vaginal delivery. It has proven to be tolerable, easy to learn and perform and having no adverse effects. it is not an exhausting maneuver. Further RCT is recommended to investigate this maneuver in comparison with other techniques as bimanual compression or aortic compression. Although this study is a single-arm type and not comparative, it has shown the effectiveness of applying the Mostafa Maged maneuver.

## Figures and Tables

**Figure 3:**
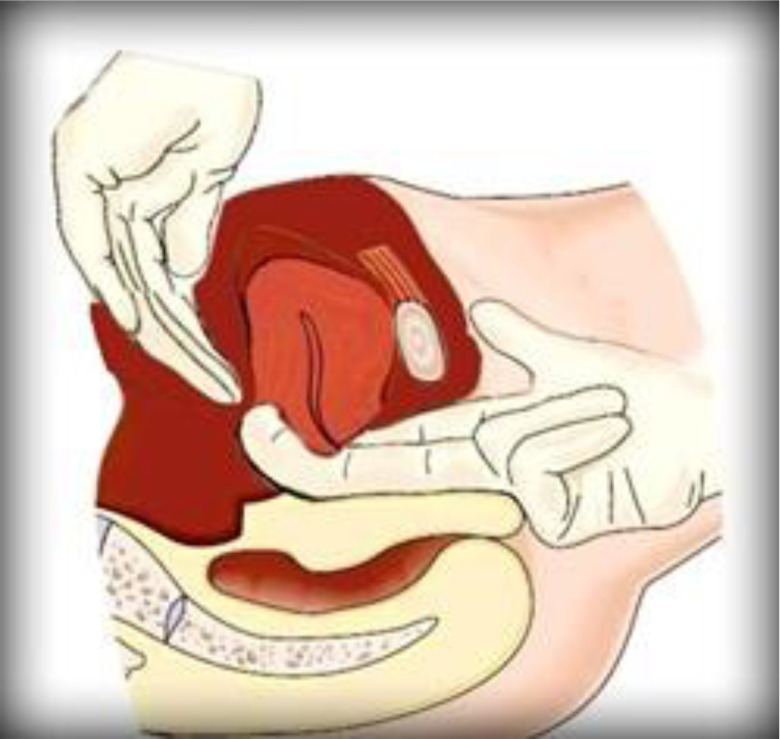
Mostafa Maged maneuver.
